# Proposal of a modified technique of Nikolsky's sign in oral autoimmune vesiculobullous diseases

**DOI:** 10.3389/froh.2024.1456385

**Published:** 2024-08-12

**Authors:** Massimo Petruzzi

**Affiliations:** ^1^ Interdisciplinary Department of Medicine, School of Dentistry, University of Bari Aldo Moro, Bari, Italy; ^2^ Department of Restorative, Preventive and Pediatric Dentistry, University of Bern, Bern, Switzerland

**Keywords:** Nikolsky’s sign, oral blisters, oral pemphigus, oral pemphigoid, oral vesiculobullous diseases

## Abstract

Nikolsky's sign, originally described for skin lesions, presents challenges when applied to the oral mucosa. To address this, a modified Nikolsky's sign has been proposed specifically for the oral mucosa. In this variant, a gentle breath of air from the air syringe embedded in the dental unit is used to inflate a pre-existing collapsed blister (non-induced technique). Alternatively, in the induced technique, a healthy peri-lesion mucosal site is gently scratched with a blunt dental tool, and after a few minutes, air is blown on the same area to inflate any newly formed blister. The sign is considered positive if a blister is raised from the blown surface. The described modified Nikolsky's sign improves the visualization of oral vesicles and blisters in a cost-effective, easy, and minimally invasive manner. Its elicitation can aid in referring patients to specialized tertiary care units.

## Introduction

Autoimmune oral vesiculobullous diseases (OVBD) encompass a diverse group of conditions, such as pemphigus, mucous membrane pemphigoid, epidermolysis bullosa, Stevens-Johnson syndrome, graft vs. host disease, and lichen planus pemphigoid (1). Differential diagnosis among these entities is challenging and requires the use of direct and indirect immunofluorescence methods, enzyme-linked immunosorbent assay (ELISA), and histopathology to establish an accurate diagnosis (1).

The presence of intact blisters or vesicles on the oral mucosa is rare compared to the skin. This rarity is attributed to the rapid rupture of blisters due to the thinner epithelium of the oral mucosa and the continuous micro-traumas caused by chewing, swallowing, and talking (2). Consequently, clinicians often observe erosive or ulcerative mucosal areas covered by epithelial flanges resulting from the collapsed blisters’ roof (3).

Blisters observed in pemphigus and pemphigoid are typically a consequence of autoantibodies targeting specific desmosome epitopes involved in intercellular connections or hemidesmosomes that anchor the basal epithelial cell layer to the basal lamina. The pro-inflammatory environment reduces cell adhesion, leading to further blister formation and vesicle development in response to minor mechanical trauma (4).

In 1896, the Russian dermatologist Piotr Nikolsky noted this phenomenon and described how applying tangential pressure to a healthy area adjacent to a lesion could induce the formation of new blisters. This observation gave rise to “Nikolsky's sign,” which was proposed to differentiate intraepidermal bullous disorders from subepidermal vesiculobullous diseases that typically do not exhibit this phenomenon (5).

In dermatology practice, eliciting Nikolsky's sign remains an essential step in the diagnostic assessment of vesiculobullous diseases. Two variants of Nikolsky's sign have been described: the “wet Nikolsky's sign” and the “dry Nikolsky's sign.” The wet Nikolsky's sign is characterized by a moist, glistening, and eroded base after pressure is applied to the skin, while the dry variant presents a dry base without serous or exudative secretions (6).

Other variations of Nikolsky's sign include the “marginal Nikolsky's sign” and the “direct Nikolsky's sign.” The marginal Nikolsky's sign is elicited by rubbing the normal skin adjacent to an existing lesion, causing the extension of erosion or blistering to the surrounding skin. The direct Nikolsky's sign is tested on unaffected skin distant from the lesions (6).

In the oral cavity, Nikolsky's sign resembles its dermatological counterpart with some distinctions. The marginal method is performed by rubbing the edge of an affected area with a dental tool, while the apparently healthy oral mucosa is gently rubbed in the direct method. The appearance of a new blister or enlargement of an existing one after eliciting Nikolsky's sign is considered positive, while the absence of blister formation is considered negative (6).

However, the wet and dry variants of Nikolsky's sign are not applicable in the oral cavity due to the presence of saliva, and detecting blisters may not always be straightforward. We describe a simple technique for assessing the presence of blisters and vesicles on the oral mucous membranes using the air syringe, which is a standard component of every dental unit.

## The technique

### The modified Nikolsky’s sign in OVBD: the induced and not induced technique

A novel technique utilizing the air syringe embedded in every dental unit is proposed to detect the presence of blisters or vesicles in the oral cavity. The operator exposes the oral mucosal site to be examined using two dental mirrors or oral retractors.

In the non-induced technique, pre-existing blisters are visualized. A gentle breath of air from the air syringe is directed tangentially to the affected mucosa to facilitate the entry of air into the pre-collapsed blister through the missing portions of the roof, which caused its collapse. The blister is inflated, and when the operator stops blowing, it collapses again. The sign is considered positive if at least one blister is observed.

In the induced technique, a peri-lesion mucosal site is gently scratched with a blunt dental tool. After a few minutes, air is blown on the same area in the same way as described before. The sign is considered positive if a blister is raised from the blown surface.

The airflow needs to be directed with a tangential trajectory across the mucous surface and not with a perpendicular one, which would drop down the blister roof instead of raising it. In clip Supplementary Video S1, the “induced sign” is demonstrated.

**Figure 1:**
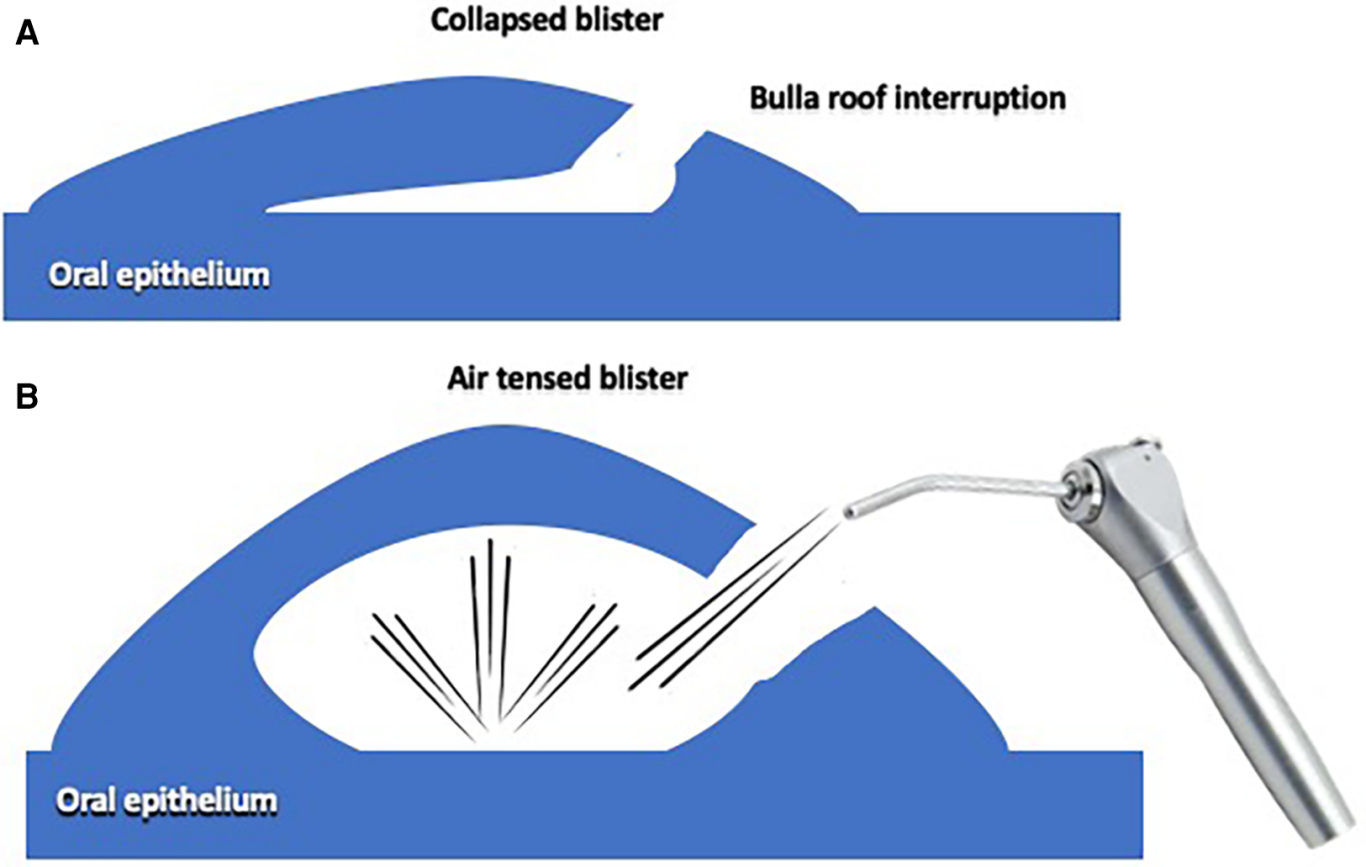
(**A**) The blister is collapsed, the roof partially broken. (**B**) The air gentle inflated by the syringe, evidence the blister, previously collapsed.

Figure 1 shows a schematic illustration of an inflated blister with the air syringe.

## Discussion

The detection and accurate diagnosis of OVBD present ongoing challenges for clinicians. Oral pemphigus and pemphigoid are rare diseases that are not commonly encountered by general dental practitioners in their routine clinical practice. As a result, there is often a significant delay in diagnosing OVBD, with studies estimating an average delay of 6–12 months (7). This diagnostic delay has important implications for treatment outcomes and the patient's quality of life (8).

In the clinical history of patients with OVBD, gingival lesions are often initially attributed to plaque-related issues and treated with scaling, root planning, and oral hygiene instructions. However, if there is a poor response to these interventions and persistent inflammation of the gingival mucosa, clinicians should consider the possibility of “non-plaque-induced gingivitis” and initiate a specific diagnostic pathway for OVBD (9).

The diagnostic pathway for OVBD typically involves ELISA testing for BP180, DSG1, and DSG3 antibodies, histopathological examination, and direct immunofluorescence, which are usually performed in specialized tertiary care units. The elicitation of Nikolsky's sign, as described, could be used as a preliminary clinical indicator of OVBD during the initial dental examination, prior to further laboratory testing (10, 11).

It is worth noting that Nikolsky's sign elicitation in the oral mucosa was first reported by Sheklakov, another Russian dermatologist (12). Subsequently, Endo et al. described the “marginal” and “direct” methods of Nikolsky's sign elicitation, involving rubbing the edge of the affected area or inducing erosion by rubbing unaffected gingiva distant from the lesions, respectively (13). Mignogna et al., using similar procedures, reported a specificity of 96.3% and a sensitivity of 46.7% for Nikolsky's sign in a study involving 566 OVBD patients (6).

Although Nikolsky’s sign was initially intended to differentiate intraepidermal blistering diseases (such as pemphigus, toxic epidermal necrolysis, and staphylococcal scalded skin syndrome) from subepidermal blistering diseases (such as pemphigoid), its application to the oral mucosa has limitations: a) it does not allow for the distinction between subepithelial and intraepithelial vesiculobullous diseases; b) it does not permit a biopsy to be taken from the area of the oral mucosa where the sign was elicited; c) in the induced sign, it could result in minimal burning symptoms in the area where the sign was provoked.

However, the described modified Nikolsky’s sign, can be useful in differentiating pemphigoid and pemphigus from similar conditions such as oral lichen planus (excluding the pemphigoid variant), oral lupus, and oral erythema multiforme.

## Conclusions

The proposed modified Nikolsky's sign, both in the induced and non-induced techniques, can be easily performed chair-side using a probe and the air syringe. The gentle insufflation with the air syringe clearly demonstrates the presence of blisters.

Performing the sign, when diagnosing a suspected OVBD, can direct the clinician to request more specific laboratory tests (ELISA, indirect immunofluorescence) and to plan a simultaneous dual oral mucosal biopsy: one for routine hematoxylin and eosin staining and one for direct immunofluorescence.

Further studies are needed to evaluate the sensitivity and specificity of the modified Nikolsky’s sign and its applicability for general dental practitioners.

## Data Availability

The raw data supporting the conclusions of this article will be made available by the authors, without undue reservation.
